# Effects of zinc supplementation forms (ZnO, Zn-Lysine, Nano-ZnO) on growth performance and metabolic health in pre-weaned Holstein calves

**DOI:** 10.1038/s41598-026-51255-x

**Published:** 2026-05-02

**Authors:** Ahmadreza Alipour, Mahdi Ganjkhanlou, Abolfazl Zali, Sadegh Hashemi, Mohammad Hasan Mortazavi, Valiollah Palangi, Wenzhu Yang, Morteza Hosseini-Ghaffari

**Affiliations:** 1 Department of Animal Science, Faculty of Agriculture, College of Agriculture and Natural Resources, University of Tehran, Karaj, Alborz Iran; 2 Department of Animal Science, Faculty of Agriculture, Near East University, Nicosia, 99138 Northern Cyprus Türkiye; 3 Lethbridge Research and Development Centre, Agriculture and Agri-Food Canada, Lethbridge, Canada; 4 Research Institute for Farm Animal Biology (FBN), Dummerstorf, 18196 Germany

**Keywords:** Antioxidants, Biochemical indices, Growth performance, Nanotechnology, Biochemistry, Biotechnology, Nanoscience and technology

## Abstract

The pre-weaning period is critical because early-life nutrition and management influence growth, metabolic function and Rumen development, thereby affecting subsequent productivity in dairy calves. Zinc (Zn) supplementation plays a key role in supporting these processes through its involvement in enzymatic activity, antioxidant defense systems, and metabolic regulation, but conventional sources often have bioavailability limitations due to the formation of insoluble complexes in the gastrointestinal tract. This study addresses this challenge by evaluating three Zn forms (ZnO, Zn-lysine, and nano-ZnO) to identify the most effective source for enhancing growth rates, nutrient utilization, and metabolic health. Twenty-four newborn Holstein calves, each with an initial body weight of 40.5 ± 4.24 kg, were selected and randomly allocated to receive one of three treatments: ZnO, Zn-lysine, and nano-ZnO supplementation. Each calf received 80 mg of Zn daily. Supplementation with nano-ZnO increased dry matter intake (*P <* 0.01), average daily gain (*P <* 0.01), and hip width (*P <* 0.01) compared to Zn-lysine and ZnO. However, there were no differences in feed conversion ratio. The treatments did not affect apparent digestibility or rumen fermentation, except for a lower rumen ammonia nitrogen concentration in the nano-ZnO group compared to the other two treatments (*P <* 0.01). Regarding blood parameters, calves receiving Nano-ZnO showed higher blood triglyceride concentration (*P =* 0.04) and superoxide dismutase activity (*P <* 0.01), while blood D-lactate concentration was lower in the nano-ZnO and Zn-lysine groups than in the ZnO group (*P =* 0.01). Additionally, both fecal consistency (*P =* 0.02) and nasal discharge (*P <* 0.01) scores were significantly reduced in the nano-ZnO group. In summary, the study suggests that nano-ZnO is a more effective Zn source and an efficient additive for improving dairy calf performance.

## Introduction

Optimizing calf growth and health is critical in dairy production, as nutritional management during the early stages of life has profound long-term effects on growth performance, metabolic development, rumen function, and ultimately future productivity^1^. To achieve this, improved nutritional strategies are increasingly employed, including the use of functional feed additives and targeted mineral supplementation^2^, given the essential roles of trace minerals as cofactors in numerous metabolic and enzymatic processes^3^. For instance, zinc (Zn) serves as cofactor for enzymes involved in immune regulation, magnesium for ATP-dependent enzymes, manganese for superoxide dismutase, selenium for glutathione peroxidases, and iron for hemoglobin, myoglobin, and cytochromes^4^. One challenge is that significantly increasing the concentration of these elements in colostrum or milk through maternal diet supplementation is not feasible^5,6^. Therefore, it may be necessary to supplement trace minerals directly in the diet of young calves^6^.

Zinc is an essential trace mineral in animal nutrition, with more than 10% of the genome encoding Zn-dependent proteins, including enzymes, signaling molecules, transport proteins, and proteins involved in DNA repair, replication, and translation^7,8^. Zn also acts as a catalytic and structural cofactor for numerous enzymes involved in protein synthesis, lipid metabolism, antioxidant defense, and immune regulation, thereby contributing to cellular and systemic homeostasis^9,10^. Zinc supplements are commonly supplied as inorganic salts, organic complexes, or nano forms^11^. Organic Zn complexes may enhance bioavailability by facilitating active absorption and reducing interactions with antagonistic compounds such as hydroxides, carbonates, phosphates, and oxalates^12,13^. Nano-minerals have attracted attention due to their small particle size and large surface area, which may enhance intestinal absorption and biological activity^14^. Several studies have shown that supplementing milk with 80 mg of Zn daily as ZnO or Zn-methionine during the first two weeks of a Holstein calf’s life improves performance and health^15,16^. It has been demonstrated that supplementing newborn calves with 32 mg/kg DM Zn as ZnO nanoparticles can effectively improve their health status during the first four months of life^17^. Some studies suggest that supplementing with nano ZnO can enhance rumen microbial activity by reducing rumen ammonia nitrogen levels through increased protein synthesis, denaturation of soluble proteins, and inactivation of proteolytic enzymes^18^. Some studies have indicated that Zn supplementation can enhance nutrient digestibility by increasing the activity of certain digestive enzymes, such as carboxypeptidase and phospholipase A2, as a cofactor^8,19^.

Despite these advances, critical gaps remain in our understanding of Zn supplementation strategies for pre-weaned calves^20^. First, although individual studies have examined ZnO, Zn-amino acid complexes, or nano-ZnO separately^10,17,21^, no comprehensive comparison of these three major Zn sources (traditional, organic, and nano) has been conducted under identical rearing conditions^10,17,21^. Second, existing research has predominantly focused on short-term outcomes (< 1 month) or isolated parameters, leaving the long-term synergistic effects on holistic calf development (e.g., concurrent impacts on growth, blood metabolites, rumen function, and digestibility) poorly characterized^22–24^. Accordingly, the present study provides a direct comparison of nano-ZnO with two commonly used Zn sources under identical rearing conditions and over a relatively extended experimental period, while simultaneously evaluating growth performance, rumen fermentation, nutrient digestibility, blood metabolic responses, and health parameters in pre-weaned Holstein calves.

Based on the improved bioavailability of nano-minerals and the chelating properties of amino acid complexes^25,26^, we hypothesized that nano-ZnO and Zn-lysine would demonstrate greater efficacy than conventional ZnO in enhancing growth performance, nutrient digestibility, and metabolic health of pre-weaned calves, due to their higher absorption efficiency and targeted enzymatic modulation^27–29^.

This study evaluated the effects of different Zn sources (ZnO, Zn-lysine, and nano-ZnO) on growth performance, rumen fermentation, digestibility, and blood metabolites in pre-weaned Holstein calves, with the aim of identifying the most effective zinc source for improving calf health and productivity during early life.

## Materials and methods

### Ethical statement

All animal procedures were approved by the Animal Care and Use Committee of the University of Tehran (ACUC Protocol #UT12-42) and conducted in accordance with the guidelines of the Iranian Council of Animal Care. The study also complies with the ARRIVE guidelines for reporting in vivo experiments, and all methods were performed in accordance with the relevant guidelines and regulations.

### Experimental design

The study was conducted at the research farm of the Faculty of Agriculture, University of Tehran, Karaj, Alborz, Iran. Twenty-four newborn Holstein dairy calves with a birth weight of 40.5 ± 4.24 kg were selected and randomly allocated to three treatments (8 calves per treatment, 4 males and 4 females): ZnO, Zn-lysine and nano-ZnO treatment. Calves received 10 mL of the respective Zn solution daily (8000 mg Zn/L; equivalent to 80 mg Zn/d). The level of 80 mg Zn/d used in this study was chosen based on previous studies^15,22,30^. This dose has been associated with improvements in growth, health, and immune-related traits without representing an excessive supplementation strategy according to recent meta-analysis conducted by ^31^. Selecting the minimum effective dose also improves the practical and economic relevance of the supplementation strategy. Zinc-lysine and nano-ZnO were selected because, together with conventional ZnO, they represent three biologically and physiochemically distinct classes of zinc source. This design was intended to maximize biological contrast among treatments rather than compare sources within the same general class. For example, hydroxy zinc, although relevant in animal nutrition, remains a mineral inorganic source with a mode of use closer to conventional zinc salts. The calves received a mixture of Zn supplement solution and milk for 61 days according to^30^.

### Zinc sources

The ZnO was purchased commercially from Merck, Germany. Zn-lysine was produced in vitro using L-lysine monohydrochloride and Zn sulfate as described previously by^32^. ZnO nanoparticles were synthesized using a precipitation method with Zn(NO₃)_2_ (Merck, Germany, 108833) and KOH (Merck, Germany, 105032) as precursors according to^33^.

### Calf management

Calves were separated from their dams immediately after birth and housed individually (2.40 m × 1.20 m) with straw bedding and concrete walls to minimize cross-contamination. Calves received 4 L of colostrum (≥ 22% Brix; according to^34,35^), using a bottle within the first 12 h of life based on^36^, and after that, 2 L of transition milk twice a day at 08:00 and 17:00 on days two and three. The calves were fed 6 L/day of raw milk from days 4 to 42, 4 L/day from days 43 to 50, and 2 L/day from days 50 to 65, at 08:00 and 17:00 using a bucket as described by^37^. A mash concentrate starter (hammer mill with a screen size of 1.95 mm) was provided to the calves ad libitum starting on day 7 as previously reported by^38^. Additionally, from day 15, alfalfa hay (particle size of 2 to 3 cm) was mixed with the starter at 10%. The timing of forage provision (day 15) to calves was determined based on a comprehensive review presented by ^39^. The starter formulation and nutritional composition of the rations are shown in Table 1. The Zn concentration of the diet was maintained at 35.5 to 36.5 mg/kg dry matter (DM). The animals had free access to water, and the solid diet was provided ad libitum throughout the experimental period. The well-being of the calves was monitored and recorded after birth and during the entire experimental period.

### Growth and intake measurements

Body weight (BW), height, and hip width were measured at 07:30 before the morning milk on days 4, 28, 42, 56, and 65. Feed intake was recorded daily by weighing the feed offered and the leftovers throughout the experimental period. Dry matter intake (DMI) was calculated from the daily feed consumption, adjusted for its DM content. Average daily gain (ADG) was calculated as the difference between initial and final BW divided by the number of days in the experimental period. Feed conversion ratio (FCR) was calculated by dividing DMI by ADG.

### Feed and fecal sampling

Fecal grab samples were collected twice daily (morning and afternoon) over three consecutive days (days 63 to 65) from each calf and stored at − 20 °C until analysis. Samples of starter and alfalfa hay were collected twice weekly and kept at − 20 °C for analysis according to^40^.

### Rumen fluid sampling

Rumen fluid was collected from the calves using a stomach tube 4 h after the first meal on day 65 based on^41^. The collected rumen fluid was strained through three layers of cheesecloth. Then, 10 mL of rumen fluid was immediately mixed with 0.2 mL of sulfuric acid solution (50% distilled water and 50% sulfuric acid) to prevent further fermentation according to ^40^.

### Blood sampling

Blood samples were collected from the jugular vein of calves using heparin vacutainer tubes before the morning feeding at 07:30 on days 28, 52, and 65. Plasma was separated by centrifugation at 3,000 × *g* for 15 min (Z 200 A, Maschinenfabrik Berthold Hermle AG, Gosheim, Baden-Württemberg, Germany) and stored at − 20 °C as reported previously by^42^.

### Health scoring

Fecal consistency, nasal discharge, eye discharge, and ear status were monitored daily as indicators of calf health. These parameters were scored using a standardized system from the University of Wisconsin, with each category rated on a scale from 1 to 4, where 1 indicated normal health and 4 represented severe symptoms (https://www.vetmed.wisc.edu/fapm/wp-content/uploads/2020/01/CalfHealthScoringChart-2018-EN-std.pdf, accessed May 2024).

### Nutrient composition and apparent digestibility measurements

Feces samples were dried at 65 °C for 48 h in a forced-air oven (Model Plus; GALLEKAMP, London, England), and then ground (FDR 90 L/4P, Dietz-Motoren GmbH & Co. KG, Dettingen unter Teck, Baden-Württemberg, Germany) to pass through a 1-mm sieve. The DM of the feed (1-mm screen) and fecal samples were determined by drying at 105 °C for 24 h (^43^ method 930.15)in an oven (Plus oven, GALLEKAMP, London, Greater London, England). Organic matter (OM) was estimated as the difference between dry weight and ash upon complete combustion of a sample at ^55^0°C for 6 h (^44^; method 942.05) in a muffle furnace (MR 170, Heraeus, Hanau, Hesse, Germany). Total nitrogen content in feed and feces was determined by acid digestion, distillation, and titration using the Kjeldahl method (KJELTEC AUTO 1030 Analyzer, Foss A/S, Hillerod, North Zealand, Denmark), and crude protein (CP) was assessed as *N* × 6.25 (^45^; method 990.03). Neutral detergent fiber (NDF) was determined by the procedure presented by^46^. The ether extract was measured with the Soxhlet method (SoxTEC System HT 1043, Foss A/S, Hilleroed, North Zealand, Denmark) using petroleum ether as solvent^47^; method 920.39) Metabolizable energy, calcium, phosphorus, and Zn values were calculated using the NASEM-Dairy-8 software^48^. Apparent digestibility in the total digestive tract was measured using the acid-insoluble ash (AIA) method^49^.

### Rumen fermentation parameters measurements

The pH of the rumen fluid was measured immediately after sampling using a portable pH meter (PP775, Zag chemie Yaran, Tehran, Tehran, Iran). To measure the Ammonia- N in the rumen fluid based on^50^, the centrifugation of samples was conducted at 3,000 × *g* for 15 min (Z 200 A, Maschinenfabrik Berthold Hermle AG, Gosheim, Baden-Württemberg, Germany). Then, 40 µL of the supernatant were mixed with 40 µL of distilled water, 2.5 mL of phenol reagent, and 2 mL of alkaline hypochlorite reagent. The mixture was then placed in a Bain-Marie at 37 °C for 10 min. The content of Ammonia-N was measured using a spectrophotometer (UV-2100, SHIMADZU CORPORATION, Seoul, South Korea) at 550 nm. Rumen Volatile Fatty Acids (VFAs) (Acetate, Propionate and Butyrate) were measured using gas chromatography by injecting 2 µL of sample into a gas chromatograph (GC-PU4410, PHILIPS, London, Greater London, England) with an FID detector, with a column length of two meters and diameter of 45 mm (10PEG), and nitrogen as carrier gas at 200 °C, the method was based on^51^.

### Blood parameters measurements

The levels of plasma glucose (Cat. No. 117500), total protein (TP, Cat. No. 130500), albumin (Cat. No. 101500), urea (Cat. No. 141500), triglyceride (Cat. No. 132500), cholesterol (Cat. No. 110500), alkaline phosphatase (ALP, Cat. No. 105500), and D-lactate (Cat. No. 114500) were measured using Pars Azmoun Assay Kits (Pars Azmoon, Tehran, Iran) and a plate reader (DANA 3200, GARNI Medical Engineering, Tehran, Tehran, Iran), following the manufacturer’s instructions. Additionally, superoxide dismutase (SOD) and zinc were assayed using kits from Navand Salamat (Cat. No. 15023, Urmia, Iran) and Biorex Fars (Cat. No. 0462, Shiraz, Iran), respectively.

### Statistical analysis

The data were analyzed using SAS software (version 9.4; SAS Institute Inc., Cary, NC, USA). Normality of residuals was evaluated using the Shapiro-Wilk test. All variables satisfied these assumptions (*P >* 0.05), justifying the use of parametric methods. The MIXED procedure Applied to repeated-measures data including growth performance (DMI, ADG, FCR and BW), skeletal development (height, hip width), and blood parameters (glucose, TP, Albumin, Urea, Triglyceride, Cholesterol, SOD, ALP, D-lactate, and Zinc) based on^52^. The statistical model used was as follows:

$$Y_{{ijk}} {\mkern 1mu} = {\mkern 1mu} \mu {\mkern 1mu} + {\mkern 1mu} b\left( {{\mathrm{IBW}}} \right){\text{ }} + {\text{ T}}_{i} + P_{j} + A\left( {Ti} \right)_{k} + {\text{ TP}}_{{ij}} + e_{{ijk}}$$

where *Y*_*ijk*_ represents the dependent variables; µ is the overall mean; b(IBW) is the covariate factor (initial weight); *T*_*i*_ is the fixed effect of the treatment; *P*_*j*_ is the fixed effect of the experimental period; *A*(*Ti*)*k* is the random effect of each animal within each treatment; TP_*ij*_ is the fixed effect interaction between the treatment and the experimental period, and *e*_*ijk*_ is the error term. Among the candidate covariance structures evaluated for repeated measures, the final fitted covariance structure was selected based on the lowest corrected Akaike information criterion (AICc) and Bayesian information criterion (BIC) values. Accordingly, the compound symmetry (CS) covariance structure provided the best fit and was used in the repeated-measures analysis performed with the MIXED procedure. Variables measured only once, including apparent digestibility (DM, OM, EE, CP, and NDF) and ruminal fermentation parameters (pH, ammonia-N, total VFA, acetate, propionate, and butyrate), were analyzed using the GLM procedure^53^. Qualitative repeated data, including fecal consistency, nasal discharge, eye discharge, and ear status, were analyzed using the GLIMMIX procedure according to^54^. Each calf was considered the experimental unit, and comparisons were made using least squares (LS) means. Gender was initially included as a fixed effect in the statistical model to account for potential sex-specific responses; however, preliminary analyses revealed no significant gender effect (*P >* 0.05). Statistical significance was set at *P* ≤ 0.05, and a trend was considered at 0.05 < *P* ≤ 0.10.

## Results

### Growth performance and skeletal development parameters

**Fig. 1:**
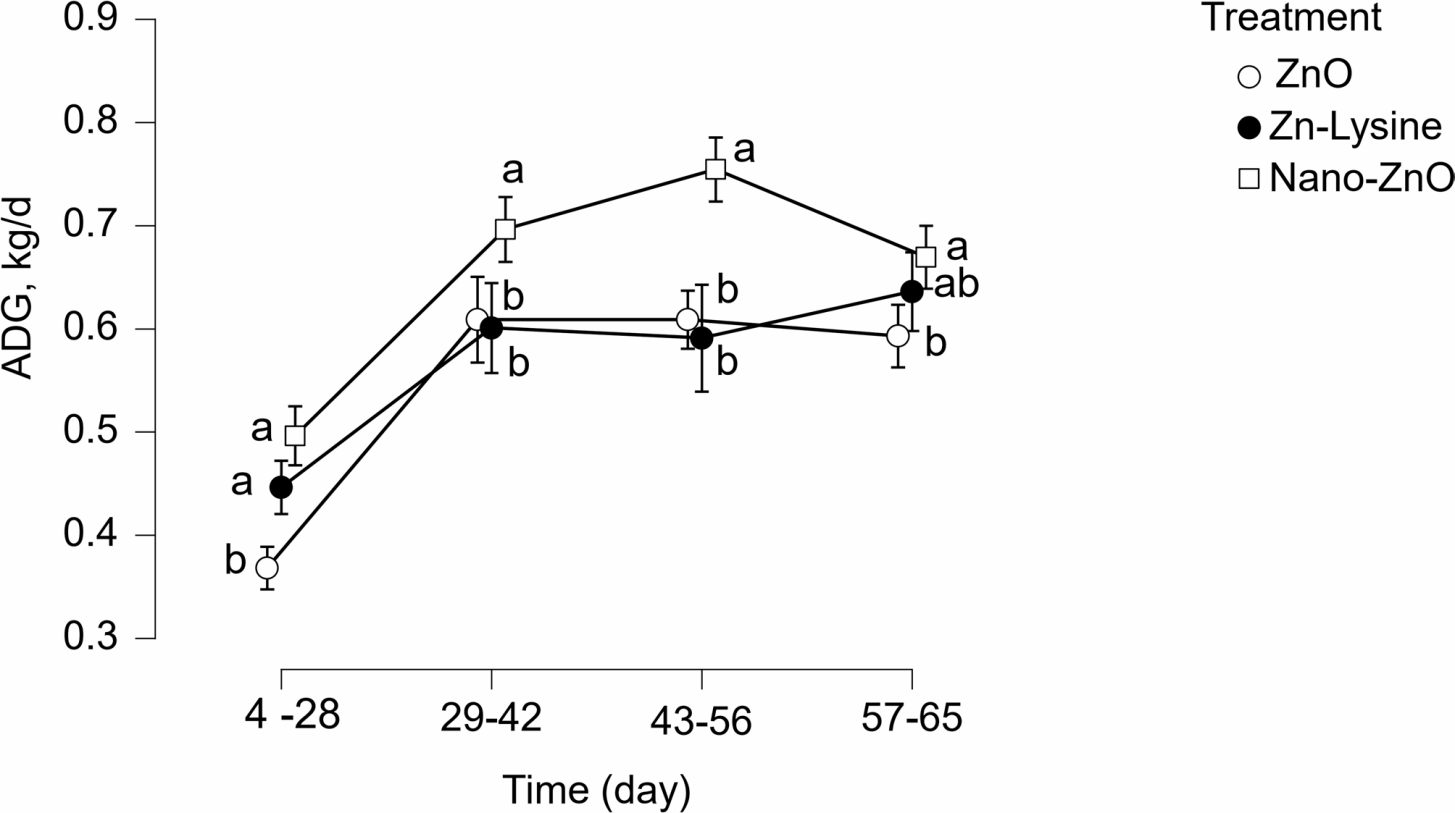
Average daily gain (ADG, kg/d) of calves over the experimental period for each treatment. Data are presented by treatment (ZnO, Zn-Lysine, Nano-ZnO) across different time intervals (4–28, 29–42, 43–56, and 57–65 days). Different letters (**a**,** b**) denote significant differences between treatments within each time period (*P <* 0.05). Error bars represent standard errors.

**Fig. 2:**
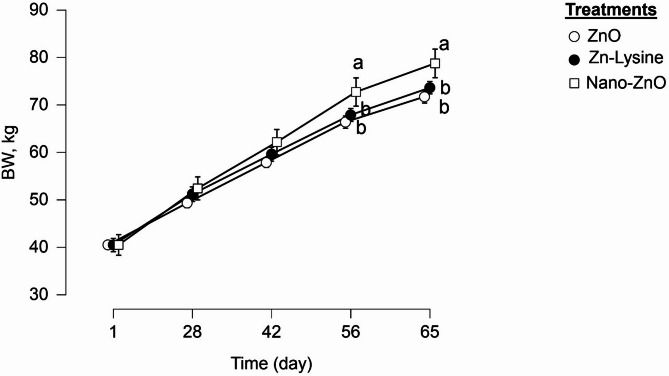
Body weight (BW, kg) of calves over the experimental period for each treatment. Data are presented by treatment (ZnO, Zn-Lysine, Nano-ZnO) across different time points (day 4, 28, 42, 56 and 65). Different letters (**a**,** b**) denote significant differences between treatments within each time period (*P* < 0.05). Error bars represent standard errors.

Data on growth performance and skeletal development are presented in Table 2. Throughout the experimental period, Nano-ZnO supplementation significantly increased ADG (*P <* 0.01; Fig. 1), DMI (*P <* 0.01), BW (*P <* 0.01; Fig. 2), height (*P =* 0.03), and hip width (*P <* 0.01). However, FCR did not differ significantly among the experimental groups (*P =* 0.37). ADG was higher in calves receiving nano-ZnO compared with those receiving ZnO and Zn-lysine, while the ZnO and Zn-lysine treatments did not differ from each other. DMI was greater in calves supplemented with nano-ZnO than in calves receiving ZnO and Zn-lysine, whereas ZnO and Zn-lysine treatments showed similar values. BW was greater in calves supplemented with nano-ZnO compared with those receiving ZnO and Zn-lysine, whereas no difference was observed between the ZnO and Zn-lysine treatments. Calves receiving nano-ZnO exhibited greater body height compared with those receiving ZnO and Zn-lysine, whereas no difference was observed between the ZnO and Zn-lysine groups. Hip width was greater in calves supplemented with nano-ZnO compared with the ZnO and Zn-lysine treatments, while the latter two treatments did not differ from each other (Table 2).

**Table 1:** Starter ingredients and nutrient composition of diet fed to the calves.

Ingredients	Content, g/kg DM
Barley grain	100
Corn grain	490
Soybean meal	280
Wheat bran	90
Fat calcium salt	10
Calcium carbonate	13
Sodium bicarbonate	5
Salt	2
Bentonite	5
Vitamin and mineral premix^1^	5
Total	1000
Nutrient	Content
Starter	
Dry matter, %	88.90
Metabolizable energy, Mcal/kg DM	3.46
CP, % DM	19.20
EE, % DM	5.93
Ash, % DM	6.10
NDF, % DM	15.70
Ca, % DM	0.77
P^3^, % DM	0.49
Zn^3^, mg/kg DM	36.50
Alfalfa hay	
Dry matter	87.65
Digestible energy, Mcal/kg DM	2.43
CP, % DM	15.00
EE, % DM	2.21
Ash, % DM	9.97
NDF, % DM	46.65
Ca, % DM	1.36
P, % DM	0.28
Zn, mg/kg DM	24.34
Starter + 10% alfalfa hay	
Dry matter	88.80
Metabolizable energy, Mcal/kg DM	3.34
CP, % DM	18.90
EE, % DM	5.59
Ash, % DM	6.40
NDF, % DM	18.50
Ca, % DM	0.82
P, % DM	0.47
Zn, mg/kg DM	35.50

^1^ Containing (per kg DM): vitamin A, 740,000 IU; vitamin D_3_, 220,000 IU; vitamin E, 14,000 IU; thiamine, 1300 mg; riboflavin, 1300 mg, niacin, 2000 mg; pantothenic acid, 2600 mg; pyridoxine 1300 mg; folic acid 100 mg; cobalamin, 14 mg; biotin, 20 mg; iron, 12000 mg; zinc, 0 mg; copper, 2400 mg; manganese, 8000 mg; cobalt, 40 mg; iodine, 160 mg; selenium, 60 mg.

**Table 2:** Effect of Zinc sources on performance and skeletal development parameters of calves.

Treatments^1^					*P*-value			
Item	ZnO	Zn-Lysine	Nano-ZnO	SEM^2^		Treatment	*Time*	Treatment × Time
DMI, kg/d	1.427^b^	1.460^b^	1.595^a^	0.3307		< 0.01	< 0.01	0.81
ADG, kg/d	0.545^b^	0.568^b^	0.654^a^	0.0172		< 0.01	< 0.01	0.49
FCR	1.893	2.051	1.946	0.0760		0.37	< 0.01	0.35
BW, kg	57.162^b^	58.572^b^	61.310^a^	0.4241		< 0.01	< 0.01	0.02
Height, cm	80.411^b^	80.045^b^	81.461^a^	0.3693		0.03	< 0.01	0.76
Hip width, cm	15.339^b^	15.458^b^	15.990^a^	0.0978		< 0.01	< 0.01	0.23

Means within a row with different superscript letters differ significantly (*P <* 0.05).

^1^ Treatment: ZnO, Zn-lysine, and nano-ZnO; each treatment supplied 80 mg Zn/d.

^2^ standard error of the mean.

DMI = dry matter intake; ADG = average daily gain; FCR = feed conversion ratio; BW = body weight.

### Nutrient apparent digestibility and ruminal parameters

Table 3 presents treatments on nutrient apparent digestibility in the entire alimentary canal and ruminal fermentation parameters. No differences in DM (*P =* 0.26), OM (*P =* 0.25), EE (*P =* 0.19), CP (*P =* 0.19), and NDF (*P =* 0.62) digestibility were observed among treatments. Ruminal pH (*P =* 0.80), total VFA concentration (*P =* 0.39), and the molar ratio of inividual VFA, including acetate (*P =* 0.11), propionate (*P =* 0.43), and butyrate (*P =* 0.30), did not vary among treatments. However, the ammonia-N concentration was significantly lower in the Nano-ZnO treatment than in the other treatments (*P <* 0.01). Ruminal ammonia-N concentration was lower in calves receiving nano-ZnO compared with both ZnO and Zn-lysine treatments, whereas no difference was observed between ZnO and Zn-lysine.

**Table 3:** Effect of Zinc sources on nutrient apparent digestibility in the total digestive tract and ruminal fermentation parameters of calves.

Treatments^1^					
Item	ZnO	Zn-Lysine	Nano-ZnO	SEM^2^	*P*-value
Digestibility, %					
DM	70.890	71.856	74.778	1.6187	0.26
OM	72.646	73.958	75.996	1.3404	0.25
EE	73.397	74.874	77.266	1.3790	0.19
CP	68.717	70.232	73.023	1.5586	0.19
NDF	37.337	39.961	41.665	3.0894	0.62
Ruminal parameters					
pH	6.386	6.223	6.033	0.3685	0.80
Ammonia-N, mg/dL	15.196^a^	14.663^a^	10.480^b^	0.7234	< 0.01
Total VFA, mM^3^	84.527	85.651	90.414	3.0073	0.39
Acetate, %	54.797	58.769	64.367	2.6835	0.11
Propionate, %	23.091	28.454	24.092	2.8780	0.43
Butyrate, %	7.631	7.756	7.307	0.1907	0.30

Means within a row with different superscript letters differ significantly (*P* < 0.05).

^1^ Treatment: ZnO, Zn-lysine, and nano-ZnO; each treatment supplied 80 mg Zn/d.

^2^ standard error of the mean.

^3^ Percentages for acetate, propionate, and butyrate denote molar proportions relative to total VFAs.

DM = Dry matter; OM = Organic matter; EE = Ether extract; CP = Crude protein; NDF = Neutral detergent fiber; Ammonia-N = Rumen ammonia nitrogen; Total VFA = Total volatile fatty acids.

### Blood parameters and health scoring

The results in Table 4 indicate that the treatments did not significantly affect glucose (*P =* 0.13), total protein (TP) (*P =* 0.49), albumin (*P =* 0.42), or urea levels (*P =* 0.64). However, supplementation with Nano-ZnO increased plasma triglyceride concentration (*P =* 0.04). Plasma triglyceride concentration was higher in calves supplemented with nano-ZnO compared with those receiving ZnO and Zn-lysine, while the ZnO and Zn-lysine groups did not differ from each other. The experimental treatments did not affect plasma cholesterol concentration (*P =* 0.68). Plasma SOD activity was higher with Nano-ZnO than with the other treatments (*P <* 0.01), while ALP activity did not differ among treatments (*P =* 0.32). SOD activity was greater in calves receiving nano-ZnO compared with both ZnO and Zn-lysine treatments, whereas ZnO and Zn-lysine did not differ from each other. Plasma D-lactate concentration was lower in the Nano-ZnO and Zn-lysine groups (*P =* 0.01), and plasma Zn concentration was not influenced by the zinc source (*P =* 0.49). Plasma D-lactate concentration was higher in calves receiving ZnO compared with those receiving nano-ZnO and Zn-lysine, whereas no difference was observed between nano-ZnO and Zn-lysine. Nano-ZnO supplementation also reduced the fecal consistency score compared to the other supplements (*P =* 0.02) and decreased nasal discharge compared to the ZnO treatment (*P <* 0.01). Fecal consistency score was lower in calves supplemented with nano-ZnO compared with those receiving ZnO and Zn-lysine, while the ZnO and Zn-lysine treatments showed similar values. Nasal discharge score was lower in calves receiving nano-ZnO compared with the ZnO treatment, whereas the Zn-lysine group showed intermediate values and did not differ significantly from either treatment. Eye discharge (*P =* 0.37) and ear status (*P =* 0.55) were not affected by the supplemental Zn sources. No mortality occurred during the experimental period.

**Table 4:** Effect of Zinc sources on blood parameters and health scoring of calves.

Treatments^1^					*P*-Value			
Item		ZnO	Zn-Lysine	Nano-ZnO	SEM^2^	Treatment	Time	Treatment×Time
Blood parameters								
Glucose, mg/dL		72.66	74.84	91.17	6.353	0.13	0.98	0.11
TP, g/dL		7.18	6.05	7.11	0.427	0.49	0.32	0.04
Albumin, g/dL		2.80	2.52	2.54	0.241	0.42	0.47	0.27
Urea, mg/dL		21.83	25.33	27.41	4.143	0.64	0.08	0.21
Triglyceride, mg/dL		11.69^b^	16.23^b^	22.26^a^	2.342	0.04	0.48	0.06
Cholesterol, mg/dL		58.48	49.20	51.26	7.654	0.68	0.23	0.22
SOD, U/dL		288.38^b^	306.97^b^	373.39^a^	8.663	< 0.01	0.01	< 0.01
ALP, U/L		166.26	187.48	239.48	33.288	0.32	< 0.01	0.38
D-Lactate, mg/dL		15.48^a^	14.35^b^	14.20^b^	0.258	0.01	0.51	0.46
Zinc, µg/dL		50.50	47.14	65.49	11.090	0.49	0.74	0.48
Health scoring								
Fecal consistency		1.12^a^	1.10^a^	1.05^b^	0.020	0.02	< 0.01	0.99
Nasal discharge		1.04^a^	1.02^ab^	1.00^b^	0.007	< 0.01	< 0.01	0.41
Eyes discharge		1.00	1.00	1.00	0.002	0.37	< 0.01	1.00
Ears status		1.00	1.00	1.00	0.002	0.55	0.50	0.46

Means within a row with different superscript letters differ significantly (*P* < 0.05).

^1^ Treatment: ZnO, Zn-lysine, and nano-ZnO; each treatment supplied 80 mg Zn/d.

^2^ standard error of the mean.

TP *=* total protein; SOD = superoxide dismutase; ALP *=* alkaline phosphatase.

## Discussion

The present study showed that the inclusion of Nano-ZnO increased DMI and growth performance in newborn calves, which contradicts several previous studies. In those studies, Zn supplementation at a dose of 80 mg/day using ZnO or zinc-methionine did not affect DMI during the first two weeks of life in Holstein calves^15,16^. The difference between this research and others may be due to variations in the duration and types of Zn used. In our study, DMI was significantly higher in calves supplemented with Nano-ZnO compared to ZnO and Zn-Lysine over the 61-day period. In contrast, Chang et al.^30^ reported no significant differences in DMI among control, ZnO, and Zn-Met groups during their 14-day trial, despite administering the same daily Zn dose (80 mg/d). This discrepancy may result from differences in experimental duration, Zn source, and calf age. Chang et al.^30^ studied neonatal calves (1 to 14 days old), a period when starter intake is minimal and gut microbiota is rapidly evolving^55^. In our study, the longer supplementation period (61 days) allowed calves to transition to solid feed, where Nano-ZnO’s enhanced bioavailability and sustained release might have promoted feed intake. A recent study in pre-weaned dairy calves similarly showed that the response to early-life zinc supplementation may vary according to Zn source, with differences reported in growth-related traits, starter intake, and health status among supplemental forms. This further supports the importance of source-dependent Zn effects during the pre-weaning period^56^. The use of Nano-ZnO in our study, compared to Zn-Met in Chang et al.^30^, could further explain divergent outcomes, as nanoparticle formulations often exhibit unique absorption kinetics and microbial interactions^57^. It has been suggested that a lower level of Nano ZnO can improve feed intake in piglets over a three-week experimental period compared to a high level of ZnO^58^. Zn is a crucial factor that affects appetite by regulating the expression of certain genes. It controls the expression of genes associated with satiety signals such as cholecystokinin (CCK) and leptin. On the other hand, Zn increases the expression of the pyruvate kinase gene, which helps clearance of metabolites and reduce satiety signals^59–61^. In addition, Zn can affect appetite through transcriptional regulation of these pathways via metal-response element-binding transcription factor-1^62,63^. Enhanced pyruvate kinase activity may also promote glycolysis and ATP production, thereby activating AMPK/Sirtuin1-dependent pathways involved in feed intake regulation^61^. Moreover, Zn may support hunger signaling by stabilizing ghrelin secretion and its orexigenic action^64^.

The greater ADG observed in calves receiving nano-ZnO is in line with previous reports showing improved average daily gain following nano-ZnO supplementation in livestock. For example, Sun et al.^11^ reported that nano-ZnO improved ADG in weaned piglets during both the first 14 and 28 days after supplementation. In contrast, Dinga et al.^65^ found no significant effect of ZnO nanoparticles on growth performance apart from feed intake. This inconsistency may reflect differences in animal species, age, experimental duration, dietary background, and nanoparticle preparation. In the present study, the 61-day supplementation period and the pre-weaning calf model may have provided greater opportunity for differences in Zn source bioavailability to be translated into measurable improvements in daily gain. Zn supports growth through several anabolic and health-related mechanisms. It can enhance IGF-1 synthesis and downstream mTOR signaling, thereby promoting nutrient sensing and muscle growth^66,67^. In the present study, nano-ZnO supplementation was also associated with improved DMI, health status, as reflected by lower fecal consistency and nasal discharge scores, greater SOD activity, and lower plasma D-lactate concentration, which may have contributed to better growth. In addition to supporting antioxidant defense, Zn may preserve gut integrity and nutrient absorption by reducing oxidative stress and inflammatory signaling^68^. Some studies in piglets have suggested that lower levels of Nano-ZnO maybe as efficacious as or even more effectual than higher levels of ZnO in improving ADG^19,69^. Zn also contributes to growth through its roles in protein synthesis, energy metabolism^70^, and the activity of digestive enzymes involved in protein and lipid utilization^71^.

Ma et al.^72^ reported that supplementing with 80 mg of Zn daily in form of ZnO and Zn-Met does not affect skeletal growth parameters, which is inconsistent with our study. This inconsistency is likely due to the shorter duration of their study (14 days) compared to our study (61 days) and the source of zinc (Organic and ZnO vs. nano-ZnO). Our findings suggest that adding Nano-ZnO can enhance the skeletal growth of calves. But to our knowledge direct comparative evidence for skeletal growth indices such as height and hip width remains limited in nano-ZnO studies so far. Zn may support skeletal growth by promoting osteoblast activity, protein synthesis, and bone mineralization, while also limiting bone resorption through modulation of parathyroid hormone-related effects^73^. In addition, Zn has been linked to osteogenesis through activation of the Wnt/β-catenin pathway, which enhances the expression of osteogenic transcription factors such as Runx2^74^. Zn may also suppress osteoclastogenesis by reducing receptor activator of nuclear factor kappa-B ligand signaling, thereby contributing to lower bone resorption^75^.

The improved pre-weaning growth performance observed with nano-ZnO likely reflects source-dependent differences in zinc bioavailability and downstream metabolic signaling rather than a simple increase in total zinc supply. Zinc is essential for DNA replication, RNA transcription, protein synthesis, and cell-cycle progression^28^. Therefore, even moderate improvements in zinc delivery to rapidly proliferating tissues during early life can translate into measurable differences in tissue accretion and growth. The smaller particle size and larger reactive surface area of nano-ZnO may enhance solubility, epithelial interaction, and absorptive efficiency relative to conventional ZnO^27,28^. Although both chelated Zn and nano-ZnO are generally more bioavailable than conventional inorganic Zn, the advantage of chelated Zn still depends on ligand type, chelation strength, and transporter-mediated uptake, and its biological response can vary markedly among products^76^. In contrast, nano-ZnO provides an additional physicochemical advantage, because its nanoscale size and high surface area can increase dissolution, absorption, permeability, and overall bioavailability beyond the limits of conventional chelation chemistry^27^. This interpretation is supported by comparative data showing that nano-ZnO improved Zn retention and intestinal absorptive surface area more than organic Zn-methionine in broilers^77^.

Several studies have shown that Zn supplementation, regardless of its chemical form (e.g., inorganic, organic, nano), can enhance nutrient digestibility. However, these studies did not report significant differences in digestibility between the Zn sources compared. This is consistent with our experimental design, which specifically evaluated differences among Zn sources (ZnO, Zn-Lysine, Nano-ZnO)^18,69,78^. Several elements are able to cause variations in reports on digestibility. These factors include the chemical characteristics of the organic Zn sources used, Zn levels in the diet, animal species, and factors that influence Zn solubility and stability in the gastrointestinal tract^79^. Rajaei-Sharifabadi et al. ^56^ demonstrated that supplementing with Zn sulfate or organic Zn did not impact nutrient digestibility in pre-weaned Holstein calves. While in another study, supplementation with 20 mg/kg organic Zn in the diet of lambs improved NDF and CP digestibility compared to Zn sulfate^80^. Additionally, it has been suggested that the impact of various trace mineral sources on nutrient digestibility greatly depends on their total concentration in the diet^81^.

Many researchers have reported that Zn supplementation generally has no significant effect on VFA proportions because Zn may have no role in their production and absorption^82–84^. In our study, we observed a decrease in the concentration of Ammonia-N with Nano-ZnO treatment. This aligns with the findings of Hosseini-Vardanjani et al.^18^, who showed that adding 40 mg/kg DM of Zn as ZnO or Nano-ZnO reduces the concentration of ruminal Ammonia-N in ewes. Furthermore, the reduction in the relative quantity of in vitro Ammonia-N by adding Nano-ZnO (200 mg/kg DM) was also reported^85^. It has been demonstrated that there is a direct correlation between improved nitrogen utilization efficiency and enhanced microbial protein synthesis^86^. This effect may be mediated, at least in part, through Zn-induced modulation of urease activity. By slowing ammonia release from urea, Zn supplementation may help maintain a more favorable ruminal ammonia profile for microbial growth and improve the efficiency of microbial protein synthesis^87,88^. This response has been attributed to interference with the nickel-dependent catalytic activity of urease, thereby reducing enzyme function^89^.

Plasma triglyceride concentration was higher in calves receiving nano-ZnO than in those receiving ZnO or Zn-lysine. A similar response has been reported in fattening lambs, in which ZnO nanoparticles significantly altered serum triglyceride concentration compared with the control groups^90^. In contrast, a recent study in crossbred calves found no significant effect of nano-ZnO supplementation on serum triglycerides. This discrepancy may be related to differences in animal type, physiological stage, feeding system, experimental duration, and nano-ZnO dose. In the present study, the combination of a longer supplementation period and a pre-weaning model may have allowed source-dependent differences in lipid digestion and metabolism to become more evident^91^. Zn may influence triglyceride metabolism through its roles in lipid digestion and lipogenesis. Zn-dependent phospholipase A2 contributes to phosphatidylcholine hydrolysis, facilitating micelle formation and intestinal lipid absorption^92,93^. In addition, Zn has been linked to the regulation of key lipogenic enzymes such as Glycerol-3-Phosphate Acyltransferase and other transcriptional pathways involved in hepatic triglyceride synthesis^94,95^.

Zn contributes to antioxidant defense through cytoprotective effects against oxidative stress and through proteins involved in Zn homeostasis, particularly metallothioneins^96,97^. Metallothioneins also possess marked antioxidant capacity and may therefore contribute to the protective effects of Zn against oxidative damage^98^. In line with our study, Liu et al.^10^ and Wo et al.^99^ reported no difference in plasma SOD activity among calves receiving different organic and inorganic Zn sources, whereas Pandey et al.^17^ observed increased plasma SOD activity following nano-ZnO supplementation. Because SOD is a Zn-dependent enzyme and is considered a functional indicator of Zn bioavailability^100,101^. The greater SOD activity observed in the nano-ZnO group may reflect improved Zn utilization. This interpretation is also supported by studies showing a closer relationship of SOD activity with tissue Zn status than with circulating Zn concentration^102,103^, making it a reliable indicator of systemic zinc bioavailability. In contrast, plasma Zn concentration did not differ among treatments in the present study, which is consistent with previous reports showing that different Zn sources may alter Zn digestibility without markedly affecting circulating Zn concentration^69^. This likely reflects the tight homeostatic regulation of Zn absorption, distribution, and sequestration, which limits the value of plasma Zn as a sensitive biomarker of Zn bioavailability under adequate Zn supply^104,105^. The lack of difference in plasma ALP activity may be expected because it is more influenced by the Zn level in the diet than by the Zn source^106^.

D-lactate is a bacterially derived metabolite and is considered an indicator of intestinal barrier dysfunction when detected in blood^107^. We found that the blood concentration of D-lactate was lower with more readily available Zn sources (Zn-lysine and Nano-ZnO treatments). Additionally, the fecal and nasal scores were lower in the Nano-ZnO treatment. In the present study, plasma D-lactate concentration was lower in calves receiving the more bioavailable Zn sources, particularly nano-ZnO, which was also associated with lower fecal and nasal scores. This interpretation is consistent with previous work showing that Zn-Met reduced intestinal permeability and D-lactate concentration in postnatal Holstein calves, indicating a protective effect of organic Zn on gut integrity and health^30^. These findings suggest improved intestinal and overall health status in calves supplemented with nano-ZnO. This interpretation is supported by previous evidence showing that metal oxide nanoparticles can exert antibacterial effects^108,109^, and that nano-ZnO supplementation reduces diarrhea incidence and improves fecal score in dairy calves^17^. Zn has also been associated with improved immune function in calves, including greater serum IgG concentration^68,110^.

Despite the promising outcomes observed with nano-ZnO supplementation, some limitations related to the broader application of nanoparticle-based additives should be acknowledged. Although nanoparticles may enhance mineral bioavailability, their physicochemical properties may also lead to dose-dependent biological responses and potential tissue accumulation if not carefully managed^14^. Current evidence generally indicates that zinc oxide nanoparticles have relatively low toxicity at appropriate dietary levels, their long-term biological and environmental effects in ruminant production systems remain insufficiently characterized^27^. Additionally, although the present study provides valuable insights into the comparative effects of different zinc sources in pre-weaned dairy calves, several limitations should be acknowledged. First, intestinal histomorphology was not evaluated in the present study. Assessing intestinal morphology could have provided complementary structural evidence supporting the observed improvements in calf health and growth performance and may help clarify potential mechanisms through which nano-zinc influences intestinal development and nutrient utilization. Second, gut microbiota composition was not characterized, which precluded a more detailed investigation of the interactions between zinc supplementation, microbial communities, and antioxidant responses. Third, the relatively modest sample size (*n* = 8 per treatment) may have limited the ability to detect subtle treatment differences and should be considered when interpreting the results. Finally, the study did not include a zinc-free control group because the primary objective was to compare commonly used zinc sources rather than evaluate zinc deficiency; therefore, the specific contribution of supplemental zinc relative to baseline dietary levels could not be fully isolated.

Future research could build on these findings by integrating intestinal morphological assessments with gut microbial analyses to better elucidate the biological mechanisms underlying the effects of nano-zinc supplementation. In addition, studies involving larger sample sizes and integrative approaches such as metagenomics or metabolomics may provide deeper insight into the interactions between dietary zinc supplementation, gut microbial communities, and host physiological responses under different nutritional and management conditions. In addition, further studies evaluating long-term safety and optimal inclusion levels are warranted.

## Conclusions

Overall, the pattern of responses suggests that nano-ZnO may support pre-weaning calf performance more effectively than ZnO or Zn-lysine. The concurrent improvements in feed intake, growth, and skeletal measurements, along with higher SOD activity, lower D-lactate, reduced fecal and nasal scores, and lower ruminal ammonia-N, indicate a more favorable physiological status rather than an isolated response. Although the effects on digestibility and most ruminal fermentation traits were limited, these findings suggest that nano-ZnO may be a useful nutritional strategy for supporting calf performance during early life.

## Data Availability

The data that support the findings of this study are available from the corresponding authors, upon request.
